# The Role of the Ubiquitin System in Eye Diseases

**DOI:** 10.3390/life15030504

**Published:** 2025-03-20

**Authors:** Sandra Carolina Durán-Cristiano, Laura de Diego-García, Alba Martín-Gil, Gonzalo Carracedo

**Affiliations:** 1 Basic Sciences Group, Faculty of Medicine, Universidad CES, Medellín 050021, Colombia; 2 Department of Biochemistry and Molecular Biology, Faculty of Optics and Optometry, Universidad Complutense de Madrid, 28037 Madrid, Spain; 3 Department of Optometry and Vision, Faculty of Optics and Optometry, Universidad Complutense de Madrid, 28037 Madrid, Spain; amartingil@opt.ucm.es (A.M.-G.); jgcarrac@ucm.es (G.C.)

**Keywords:** proteasome, ubiquitin, ocular surface, retinal diseases, immunoproteasome

## Abstract

The ubiquitin–proteasome system (UPS) is a fundamental process that regulates various biological functions, including immune response, cell cycle, oxidative stress, migration, and cellular proliferation. This system is responsible for the degradation of proteins, while proteasomes play a significant role in mechanisms involved in health and human diseases. The participation of the UPS in immune response is particularly relevant, leading to the involvement of immunoproteasomes. This specialized proteasome is involved in the processing and presentation of antigenic peptides, making it crucial for proper immune function. Moreover, the impact of the UPS is considered essential in understanding several diseases, such as neurodegenerative disorders, infections, and vascular diseases. The dysregulation of the UPS may contribute to the pathogenesis of these conditions, highlighting its importance as a potential therapeutic target. Interestingly, the UPS is also related to ocular structures, playing a role in visual perception and ocular homeostasis. This involvement in the regulation of various ocular processes suggests its potential impact on both anterior and posterior eye pathologies. This review aims to discuss the general considerations of the UPS and provide information about its participation in anterior and posterior eye pathologies. By understanding its role in ocular health and disease, researchers and clinicians may explore novel therapeutic strategies targeting the UPS for the treatment of various eye conditions. In conclusion, the UPS is a crucial player in biological processes, with far-reaching implications in health and disease, including the anterior and posterior segments of the eye. Further research in this field may lead to the development of innovative therapies and a better understanding of the complex mechanisms underlying various eye disorders.

## 1. Introduction

The ubiquitin–proteasome system (UPS) is one of the most specialized and critical systems in mammals, playing a profound role in protein degradation and essential cellular processes such as cell cycle regulation, immune responses, and signaling pathways [1,2,3,4]. The UPS operates through two key components: ubiquitination, a post-translational modification that regulates various cellular events, and the proteasome, a multi-subunit complex responsible for recognizing polyubiquitinated proteins and initiating their degradation [5,6].

Due to the participation of the UPS in different biological processes in the cell, this system has been a therapeutic target in many diseases such as multiple myeloma, lung cancer, autoimmunity, peripheral neuropathy, and neurodegenerative disorders [7,8].

Cell death and cellular degeneration may arise from imbalances in cellular systems or biological processes, which are associated with various ocular pathologies that present with distinct clinical manifestations. Among these events, the role of oxidative stress has been considered in recent years [9]. For example, its impact on the levels of reactive oxygen species (ROS) that promote lipid peroxidation and subsequent mitochondrial damage, reflected in pathologies such as glaucoma, macular degeneration, diabetic retinopathy, keratoconus and dry eye, suggests that oxidative stress may be involved in pathophysiology [9] and is, in turn, influenced by the activity of the UPS [10,11,12,13].

Recent research has highlighted the significant role of the UPS in a variety of diseases that disrupt cellular homeostasis, particularly in anterior and posterior ocular pathologies. These conditions share common biological mechanisms involving ubiquitin–proteasome activity, which is vital for maintaining cellular proteostasis.

This review aims to provide a comprehensive overview of the UPS and its involvement in specific ocular pathologies. Additionally, we will examine the immunoproteasome and its influence on ocular mechanisms across various physiological processes, thereby enhancing our understanding of diverse ocular diseases.

A systematic literature search was conducted using databases such as PubMed, Scopus, and Google Scholar. Keywords related to ocular diseases, treatments, ubiquitination and proteasome inhibitors were used to identify relevant studies. Articles were initially screened based on titles and abstracts to assess their relevance to the research topic. Full-text articles were then reviewed to ensure they met the inclusion criteria. The following inclusion criteria were applied: (1) studies published in peer-reviewed journals, (2) studies published in English, (3) studies focusing on ocular diseases and the relationship between UPS and eye, and (4) studies conducted to capture the most recent developments in the field. Exclusion criteria included: (1) studies not related to ocular diseases, (2) studies with incomplete data or unclear methodologies, and (3) sources that did not provide primary data (e.g., opinion pieces or editorials).

## 2. The Ubiquitin–Proteasome System

### 2.1. Ubiquitination

Proteins play a crucial role as regulatory elements in numerous essential biological processes in living beings and, in some cases, it is necessary for certain chemical modifications. These modifications, known as “post-translational modifications” (PTMs), serve to determine the catalytic activity of proteins, as well as modify their function and localization within the cell. These PTMs can involve the addition of functional groups, such as acetyl, phosphate, or methyl groups, to specific amino acid residues within the protein. Alternatively, PTMs can include the removal of certain groups or the formation of disulfide bridges between cysteine residues [14].

By altering the catalytic activity, stability, interactions, or localization of proteins, PTMs play a crucial role in regulating numerous biological processes, such as cell signaling, gene expression, metabolism, and immune response [15]. Dysregulation of PTMs has been implicated in various diseases, including cancer, neurodegenerative disorders, and autoimmune diseases. Understanding the complex interplay of PTMs and their impact on protein function is an active area of research in biology and medicine with potential applications in the development of targeted therapies and biomarkers for disease diagnosis and prognosis [16].

Among the most common PTMs that proteins undergo are palmitoylation, phosphorylation, glycosylation, methylation, and ubiquitination. Ubiquitination is a process that the vast majority of eukaryotic cells present, characterized by the covalent attachment of one or more ubiquitin (Ub) molecules to a target protein, leading to changes in its function, location, structure, or activity [17]. Ub is a small yet essential protein, composed of 76 amino acids, that plays a critical role in regulating various cellular processes. Its high conservation across eukaryotic species underscores its fundamental importance in cellular function. Covalent binding serves as a significant signal for protein degradation, particularly within the context of targeted protein degradation by proteasome.

In 2004, scientists Hershko, Ciechanover, and Rose were awarded the Nobel Prize in Chemistry for their contributions to in vitro proteolysis, where they elucidated the role of Ub in protein degradation [18].

**Figure 1:**
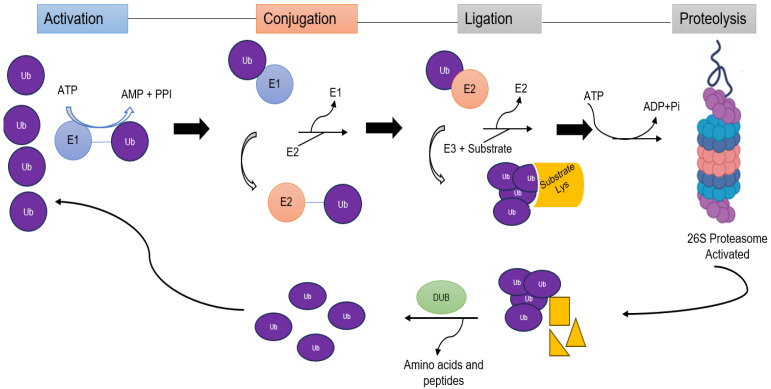
The Ubiquitin–Proteasome System (UPS). Ubiquitination is a post-translational protein modification that occurs in three key steps: activation (E1), where ubiquitin is activated; conjugation, in which ubiquitin is transferred to a ubiquitin-conjugating enzyme (E2); and ligation, where ubiquitin is attached to a substrate protein through the action of an E3 ubiquitin ligase. The polyubiquitinated protein is then recognized by the proteasome and degraded into small peptides. Deubiquitinating enzymes (DUBs) can remove the ubiquitin, allowing it to be recycled for further rounds of ubiquitination.

For the ubiquitination process to occur, the activity of several actors is required in a three-step sequential manner: first, a Ub molecule is activated by the activating enzyme or E1 in an ATP-dependent manner; then, a conjugating enzyme or E2 transports the Ub to the substrate protein; and, finally, it culminates in the formation of an isopeptide bond between the terminal α-carboxyl group of Ub and the ε-amino group of a lysine (K) present in the target protein, a function carried out by E3 ligases [10,19] (Figure 1).

Certainly, one of the aspects crucial to the diversity and intricacy of ubiquitination is the activity of the E3 ligase enzyme. Once bound and loaded with the substrate, the E3 ligase directs and determines the biological function of the modified protein (substrate). In the human genome, 2 genes have been identified as coding for E1, 40 for E2, and over 600 for E3, which demonstrates the diversity and complexity in the functions of ubiquitination [20]. The E3 ligase are classified into four types according to its structure and function: HECT type, U-box type, RBR type, and RING type [21].

The ubiquitination process, unlike other PTMs, is more complex; it involves the binding of a Ub molecule to the target protein (monoubiquitination), but also the presence of seven lysine residues (K6, K11, K27, K29, K33, K48, and K63) in the Ub enabling the formation of various polyubiquitin chains (polyubiquitination). In this case, the initially inserted Ub acts as the “acceptor or mark” for the insertion of additional Ubs. The type of linkage between the Ub molecules determines the route or destination of the ubiquitinated protein [22]. This diversity makes ubiquitination a complex process influencing multiple cellular and molecular processes [23].

Proteomic analysis has revealed that ubiquitination can occur in both cytosolic proteins located in the membrane and the cell nucleus [23]. In fact, it is well known that ubiquitination regulates a wide range of cellular processes, including cell cycle progression, endocytosis, intracellular trafficking, DNA repair, transcription, signaling and antigen presentation in the immune response, among others [7,24,25]. For instance, Jarome et al. demonstrated that ubiquitination induces changes even in gene expression by modifying histones such as histone H2B, thereby facilitating trimethylation of lysine 4 H3 in genes associated with hippocampal learning [26]. Consequently, Collins P.E. et al. described the importance of ubiquitination in the transcriptional activity of NF-κB (nuclear factor kappa enhancer in the light chain of activated B cells), the principal regulator of the immune response [27].

Just as cells have machinery that provides the insertion of Ub, and this modification triggers a cellular response, there exists a mechanism to remove Ub from proteins, thus reversing the Ub action. This process is referred to as *Deubiquitination* (DUB), and enzymes possessing this capability play a crucial role in the process. In humans, approximately 100 DUBs have been described. Generally, DUBs bind to the isopeptide bond, counteracting the Ub ligase activity and disrupting the regulation by Ub [28]. Consequently, the functions of DUBs include the following: editing Ub chains, recycling Ub molecules during the ubiquitination process, processing Ub precursors and their significant role in regulating proteasomal activity is well-documented [29,30].

### 2.2. Proteasome

The proteasome is a complex with protease activity that requires energy, predominantly found in eukaryotic cells, and is involved in the cellular machinery that modulates a significant portion of protein degradation. Activation of the proteasome via ubiquitination signaling necessitates polyubiquitination of residue 48, which serves as cue to initiate degradation through the proteasomal pathway [31,32,33]. In contrast, if the chain is formed through lysine 63, it does not act as a signal for degradation; instead, it is involved in various non-proteolytic functions, including transcription activation, protein kinase activation, DNA repair and replication, and intracellular trafficking [34,35].

Once the substrate has been marked for degradation, the proteasome, in its 26S conformation and with a molecular weight of approximately 2500 kDa, functions as a large multicatalytic protease. It is composed of the following two main complexes: the 20S core complex (the catalytic core) and the 19S regulatory complex [36]. The 20S catalytic complex features two outer α rings and two inner β rings, each comprising seven subunits that provide structural integrity (α1–α7 and β1–β7). The inner β rings exhibit the following distinct catalytic activities: post-glutamyl (β1) activity, which resembles that of caspases and cleaves substrates at acidic residues; trypsin-like (β2) activity, which targets basic residues; and chymotrypsin-like (β5) activity, which cleaves hydrophobic residues [37]. This intricate structure allows the proteasome to efficiently process and degrade ubiquitinated proteins, thereby playing a crucial role in cellular homeostasis.

The 19S complex is located at the end of the 20S center, composed of the following two structures: the base and the lid. The entry to the proteolytic chamber is typically obstructed by the N-terminal ends of the α subunits [38]. The base is the regulatory subunit (19S), which is responsible for binding, deubiquitination, unfolding, and translocating substrates to the catalytic core. It is formed by six subunits with ATPase activity that belong to the AAA family (Rpt1-Rpt6) and three subunits, namely Rpn1 and Rpn2 (scaffolding proteins) and Rpn10 (a ubiquitin receptor), lack ATPase activity. These subunits link the cap and the base of the proteasome complex and play a crucial role in stabilizing it [39]. The ATPase subunits meet the α subunits of the 20s complex [40]. The lid is formed by nine non-ATPase subunits (Rpn3, 5-9, 11, 12, and small adhesive proteins SEM1) [1,41]. Both substrate recognition and unfolding are energy-dependent processes, relying on the ATPase activity of the subunits to provide necessary metabolic energy [42,43].

In addition to the 19S regulatory complex, there are other lesser-known activating complexes, including the 11S complex (also referred to as PA28/PA26/REG) and the PA200 complex [44]. Unlike the 19S complex, both the 11S and PA200 complexes lack ATPase activity and do not unfold proteins. However, they are capable of inducing the opening of the 20S channel, thereby facilitating substrate access [45].

Proteolysis is conducted through different steps that involve the recognition of the substrate, developing translocation and deubiquitination. The substrate protein must have at least four Ubs attached to be recognized. Rpn10 is associated with polyubiquitin while Rpn1-Rpn2 binds to the protein.

Deubiquitinating enzymes separate the Ubs and the subunits with ATPase activity, using the energy of ATP, they produce the unfolding of the protein and make it pass towards the inner chamber of the central particle through conformational changes in the alpha subunits that obstruct the entry [46]. As the protein traverses the chamber, hydrolysis of the peptide bonds takes place, resulting in the release of peptides through the regulatory particle [41]. The proteasome degrades the substrate into fragments, which are subsequently processed into smaller peptides of uniform size. These peptides are then further degraded into free amino acids by the action of endopeptidases or exopeptidases, ultimately leading to their breakdown.

The proteasome is extensively documented to be involved in numerous pathological processes, including cancer, inflammatory conditions, and neurodegenerative diseases [47,48,49]. Its role in these diseases underscores the importance of the UPS in maintaining cellular homeostasis. Disruptions in proteasomal function can lead to accumulation of damaged or misfolded proteins, contributing to cellular dysfunction and disease progression. In particular, the UPS has been implicated in the pathogenesis of neurodegenerative disorders such as Alzheimer’s and Parkinson’s disease, where the failure to degrade ubiquitinated proteins results in toxic aggregates that exacerbate neuronal damage [50,51,52,53]. Understanding the mechanisms by which the proteasome influences these pathological states is crucial for developing therapeutic strategies aimed at restoring proteostasis and mitigating disease effects.

### 2.3. Immunoproteasome

The immunoproteasome is a specialized variant of the proteasome that plays vital roles in both health and disease. It is instrumental in immune responses through several mechanisms. Notably, it generates peptides for presentation by Major Histocompatibility Complex (MHC) class I molecules, a process essential for activating CD8+ T cells in response to viral infections [54,55]. The immunoproteasome differs from the standard proteasome in its peptide cleavage properties, which enhances its ability to efficiently produce antigenic peptides. Furthermore, it supports the differentiation of pro-inflammatory T helper cell subsets, such as Th1 and Th17, and facilitates the production of pro-inflammatory cytokines, including interferons, TNF-alpha, IL-6, IL-17, and IL-23 [56,57]. These cytokines contribute to the development and persistence of autoimmune diseases. Consequently, targeting and inhibiting the immunoproteasome presents a promising therapeutic strategy for managing autoimmune conditions and influencing viral infections [58].

Hematopoietic origin cells or cells stimulated with pro-inflammatory agents leads to the replacement the constitutive catalytic subunits of the proteasome (β1, β2, β5) with the inducible catalytic subunits of Low-Molecular-Weight Peptides LMP2 (β1i) and LMP7 (β5i), or with the Multi-Catalytic Endopeptidase Complex-Like MECL-1 (β2i) during proteasomal neosynthesis, forming what is known as the immunoproteasome [59,60]. Under inflammatory conditions, the 19S regulatory subunit is also substituted by the 11S subunit (also called PA28), which consists of two subunits, PA28α and PA28β. This complex is significantly smaller [61] and its involvement has been studied in various neurodevelopmental disorders, including epilepsy [62].

Furthermore, the immunoproteasome is implicated in a range of diseases. For example, its dysfunction has been associated with cardiovascular and pulmonary conditions, including chronic obstructive pulmonary disease [63,64,65]. For instance, the immunoproteasome is continuously expressed in alveolar macrophages and is quickly activated in respiratory cells during viral infections, highlighting its role in antiviral immunity in the lungs [66,67,68]. Additionally, the immunoproteasome is involved in the degradation of oxidatively damaged proteins, particularly under conditions of stress and disease [57,69]. Given these roles, it is plausible to consider its involvement in ocular pathologies, where dysregulation of the immunoproteasome has been linked to diseases characterized by protein aggregation.

In summary, the immunoproteasome is essential for antigen presentation, immune regulation, and protein homeostasis. Its dysregulation has been associated with various diseases, making it a potential therapeutic target and biomarker for disease prediction and progression.

## 3. Role of the UPS in Eye

Advancements in biology have significantly enhanced our understanding of the pathophysiological events underlying ocular diseases, including the critical roles played by specific metabolic pathways and signaling cascades. These discoveries have provided deeper insights into the mechanisms driving various ocular conditions, thus paving the way for more targeted interventions in diagnosis and treatment [70]. A notable example is the potential involvement of the UPS, whose association with protein accumulation may play a pivotal role in the pathogenesis of ocular diseases. In fact, recent studies suggest that UPS plays a key role in retinal development, photoreceptor survival, and neuroinflammation (REF). These functions are crucial in eye diseases such as glaucoma and ocular surface disorders, where inflammation and cell death are prominent.

Currently, for the management of various anterior segment ocular pathologies, pharmacological and surgical treatments are available to minimize the risk of vision loss, although their effectiveness is limited in some cases [71]. However, the development of novel therapies that can intervene at the early stages of biological processes, such as inflammation, oxidative stress, or apoptosis, holds the potential for more effective treatment prior to the manifestation of clinical signs and symptoms. One example of this is the use of proteasome inhibitors, which have been proposed as a therapeutic option in diseases like cancer. Additionally, the dysregulation of enzymatic activity in the ubiquitination process could be a key factor in the treatment of various ocular pathologies.

**Figure 2:**
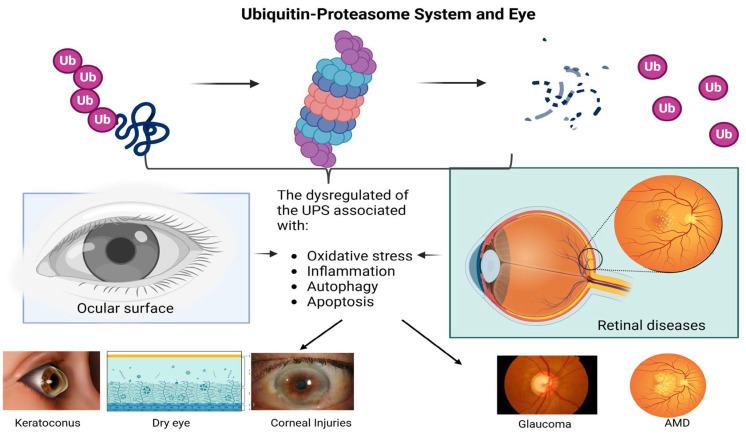
The UPS regulates key biological processes such as inflammation, oxidative stress, and cell death. Its dysfunction could be involved in anterior segment ocular diseases, such as dry eye and keratoconus, which are associated with chronic inflammation and abnormal protein degradation. In the posterior segment, the UPS may influence conditions such as glaucoma and age-related macular degeneration (AMD), where impaired protein degradation and stress response contribute to disease progression.

In Drosophila Melanogaster, ocular development requires ubiquitination to activate retinal morphogenesis and photoreceptor cells viability [72]. Furthermore, neuroinflammation plays a crucial role in glaucoma, as inflammatory mediators influence cellular responses in the retina, ultimately leading to cell loss via apoptosis. Moreover, the regulation of ubiquitination activity is vital in various ocular surface diseases, as it is maintains cellular homeostasis and influence disease progression [73,74]. However, the role of the UPS in ocular pathologies is not so widely studied, considering that the multiple roles of the UPS include regulation of inflammation, so it could be thought that some biological phenomena involved in pathologies of both the anterior segment and the posterior segment of the eye could be explained by the Ubiquitin–Proteasome pathway (Figure 2).

In this section, we discuss the role of the UPS in both anterior segment pathologies (such as dry eye and keratoconus) and posterior segment pathologies (including macular degeneration and glaucoma), highlighting its clinical relevance in the diagnosis and treatment of these conditions.

### 3.1. Anterior Segment of the Eye

#### 3.1.1. Dry Eye

Dry eye disease (DED) is a multifactorial disease that results in the loss of homeostasis of the Lacrimal Functional Unit (LFU), causing ocular symptoms and signs that, in some cases, result in visual impairment and loss of quality of life [75]. In recent decades, significant contributions from molecular and cellular biologist has allowed us to understand and establish the various events involved in the pathogenesis of the disease. Such is the case for inflammation, where once various transcription factors such as NF-κB are activated, they modulate the gene expression of cytokines such as IL-6, IL-22, and tumor necrosis factor (TNFα) and this leads to an inflammatory process [76] that modifies tear film, corneal, and conjunctival stability.

Key biological events in dry eye disease include hyperosmolarity, instability of the tear film, loss of homeostasis, inflammation, and neurosensory abnormalities [77]. These events are closely related to changes in tear quality, which in turn disrupt the LFU. This dysregulation may be associated with alterations in the proteins on the ocular surface, which can accumulate and induce apoptosis in conjunctival, epithelial cells, and Meibomian glands. Therefore, dysfunction in the UPS could lead to a loss of protein balance, potentially inducing remodeling of the ocular surface.

Accordingly, experimental studies suggest that inflammation plays a role in the diagnosis and treatment of dry eye. Thus, Kuklinski E.J. et al., demonstrated in vitro that dendrite cells (DC) are activated, and these initially mediate the activation of the innate and adaptive immune response on the ocular surface in a dry eye model [78]. However, it is necessary to recognize that antigen presentation by DC can be carried out by MHC class I and II. Although a large participation in DED is associated with the activity of MHC class II and its mechanism which modulates the activation of Th1 lymphocytes, and thus the secretion of pro-inflammatory cytokines such as TNF, IL-2, IL-17. In the case of recognition by MHC class I, once DCs are activated, they induce the production of A and B proteins related to MHC class I, which activate NK cells, and these produce IFN-γ, which leads to conjunctival and corneal epithelial activation, causing activation of Th1 cells. Interestingly, recognition by MHC class I requires proteasomal and immunoproteasome activity [59].

Recently, Xie M. et al. (2022) demonstrated, in C57BL/6 mice with dry eye and stimulated with acacetin, a natural flavone, a reduction in the activity of the NLRP3 inflammasome, and, thus, a decrease in corneal staining and an increase in the number of goblet cells in the conjunctiva. Interestingly, acacetin inactivated the NLRP3 signal through its ubiquitination, where glycoprotein 78 (gp78), known as E3 ligase, suppressed the activation of NLRP3 and attenuated the inflammatory response, suggesting a promising therapeutic target, where ubiquitination activity is involved [79].

Inflammation plays a key role in the immunopathology of dry eye, with emerging therapies focusing on suppressing this response. A critical factor in ocular surface inflammation is nuclear factor kappa B (NF-κB) [80], which promotes the secretion of pro-inflammatory cytokines [81]. The activation of NF-κB depends on the UPS, which labels IκB with Ub for degradation. Once IκB is degraded, NF-κB translocates to the nucleus and activates inflammatory gene transcription. Consequently, studies suggest that proteasome inhibitors could help reduce inflammatory mediators in diseases where inflammation is a central factor [27,82].

On the other hand, IL-17 has been studied as a therapeutic target promoter in dry eye. Alam J. (2022) established elevated levels of both IL-17 and IL-23 in corneal and conjunctival tissues in an animal model with receptor-deficient nuclear RXRα. In transcriptomic analyses, they found that ubiquitination was involved within the pathways related to IL-17 signaling [83]. This aspect is consistent with what was published by Rong Z. et al. (2007), who found that TRAF mediates the ubiquitination of IL-17R induced by IL-17F, and that its E3 ligase activity was necessary for said signaling [84], the role of residue 48 in polyubiquitination is considered crucial in determining the specific type of dry eye condition that involves the Th17 phenotype [85].

Dry eye is associated with other ocular disorders. During the process of aging and cellular senescence, ocular structures undergo important changes, especially those of the ocular surface. Nättinen J. et al. (2019) examined the effects of aging on the tear proteome, finding a relationship between age and several tear proteins related to cell death and inflammatory response, and several proteins of the ubiquitination complex [86].

In addition, ocular manifestations of limbal stem cell deficiency (LSCD) include dry eye. In an LSCD model, an increase in inflammatory cells and the activity of immunoproteasome formation with a decrease in constitutive proteasome formation was found [87,88]. Furthermore, the immunoproteasome has been implicated in the pathophysiology of dry eye disease, particularly in conditions such as Sjögren’s syndrome [89]. For example, the dysregulation of immune responses, including those mediated by the immunoproteasome, can lead to the destruction of lacrimal glands and reduced tear production, further aggravating dry eye symptoms [90]. The above suggests that it may lead to rapid immunoproteasome response and reduce constitutive proteasome activity, a change in the proteasome population after the immune response.

In studies of chronic inflammation, constitutive proteasome activity has been shown to decrease and immunoproteasome activity to increase. Therefore, in chronic stages of dry eye, it is possible that the activity of the constitutive proteasome may be affected, leading to the non-degradation of proteins via the proteasome, and, therefore, this leads to the accumulation of proteins, which subsequently contributes to oxidative stress, activation of autophagy, and subsequent cell death [91,92,93]. Thus, given its involvement in inflammation and immune regulation, the immunoproteasome is being explored as a potential therapeutic target for dry eye treatment, especially in autoimmune contexts, and as focusing on inhibitors of the immunoproteasome may help mitigate the inflammatory responses associated with dry eye conditions.

#### 3.1.2. Keratoconus

Keratoconus (KC) is a degenerative disease of the cornea characterized by cone-like steepening and irregular stromal thinning, which distorts vision. In recent years, attention has been focused on the role of inflammation as a regulator of the disease’s development and progression [94]. Understanding the pathophysiology of KC, from a molecular perspective, allows us to recognize the clinical significance of biological events such as inflammation, oxidative stress, alterations in the extracellular matrix, and the activation of apoptosis [95].

For many decades, inflammation was thought to be irrelevant in KC. However, experimental studies have demonstrated its influence on specific signals that contribute to cellular stress, alterations in the extracellular matrix, and the activation of apoptotic pathways, ultimately leading to damage in keratocytes and fibroblasts at the corneal level [96]. Nonetheless, the role of the UPS in protein regulation and stability at the corneal level remains underexplored.

The corneal epithelium has the capacity to express K3/K12 keratins and maintains regular functions related to the renewal process in the face of an injury [88]. For this, it is necessary to modulate the amount of keratin present in affected tissue and ensure that it does not accumulate and is not added at undesirable times. In addition, the constitutive proteasome plays a crucial role in protein degradation, as highlighted by Chan J.K.L. et al. (2018), who emphasized the importance of keratin 6a (K6a) and its post-translational modification by Ub. This modification influences proteasomal processing to produce antimicrobial peptides derived from keratin, which contribute to the innate immune response in the corneal epithelium, particularly in cytoskeletal remodeling in UPS-mediated infections [97]. This underscores the system’s role in generating antigenic fragments during immune responses.

Bardag-Gorce et al. characterized the proteasome in corneal epithelial cells [87] and later investigated its effects in an animal model following corneal injury. They found that the epithelial keratins K3 and K13 are polyubiquitinated for proteasomal degradation. However, corneal injury leads to impaired proteasome activity, resulting in keratin aggregation and subsequent UPS activation [88]. This suggests that proteasome activity is crucial for the formation and removal of corneal keratin aggresomes, with potential therapeutic implications for ocular surface injuries such as Limbal Stem Cell Deficiency (LSCD) and other conditions that promote corneal opacity, including keratoconus.

In addition, Ferrington D.A. et al. (2013) proposed that the immunoproteasome is key in corneal healing. Following the induction of inflammatory cytokine secretion, the constitutive proteasome becomes unstable, and the immunoproteasome is rapidly activated. This activation enables the production of peptides that are ultimately bound to MHC class I molecules [98].

On the other hand, Lomako J. et al. (2010), highlighted the importance of the proteasome in regulating MUC4, a membrane mucin involved in eye protection. They demonstrated that TGF-β induces the degradation of MUC4 in the basal and intermediate layers of the cornea, and that this effect is reversed by proteasome inhibitors (MG132), leading to an increased expression of MUC4 on corneal tissue [99]. This suggests that proteasome inhibitors could be a promising therapeutic approach for treating corneal injuries and stabilizing mucin in cases of ocular surface damage. Additionally, recent omics-based studies of corneal tissue have revealed that the UPS pathway plays a role in keratoconus (KC), with its upregulation potentially contributing to KC progression [100,101]. Thus, targeting the UPS may offer a novel therapeutic strategy for the treatment of KC.

Indeed, several studies in transcriptomics and proteomics affirm that hypothesis. Shinde V. et al. (2019), evaluated corneas of healthy individuals and those with keratoconus using mass spectrometry, detecting a total of 3132 proteins, of which 627 were over- regulated in ectatic corneas. Interestingly, within these proteins, some E3 ligases (CUL2, CUL3 and CUL4B), SUMO3 and UBA6 [100] were involved in the proteasomal pathway, among them some E3 ligases (CUL2, CUL3 and CUL4B), which could in the future be used as new therapeutic targets.

Cellular proteases, particularly those such as metalloproteinases (MMP), play a critical role in the degradation compression of the extracellular matrix and corneal thinning [102]. Several factors regulate the activity of these enzymes, with vascular endothelial growth factor (VEGF) being a prominent example, as it is expressed in both corneal epithelial and endothelial cells. Once activated, VEGF stimulates the expression of MMP-2 and MMP-9 in the cornea, leading to inflammation, scarring, and loss of corneal transparency.

Consequently, antiangiogenic factors can modulate the activity of VEGF; thrombospondin-1 (TSP-1) serves as a notable example. Bardag-Gorce F. et al. demonstrated, in cultures of epithelial cells within a neovascularization model, that TSP-1 levels decreased. However, when the proteasome was inhibited, TSP-1 levels increased, while VEGF decreased [103]. This finding is clinically relevant for the development of corneal neovascularization with proteasome inhibitors, potentially reducing visual loss.

The immunoproteasome is abundantly expressed in the corneal epithelium and plays a crucial role in maintaining corneal integrity and function. The immunoproteasome has been implicated in the pathogenesis of keratoconus, though its exact role is still being investigated. For example, immunoproteasome deficiency leads to increased apoptosis in corneal epithelial cells and delayed wound healing in mouse models [87,98].

Recently, its function has been involved in regulating inflammation, with findings suggesting a significant role in mediating oxidative stress responses [104] may be relevant in keratoconus pathogenesis. Further studies are essential to elucidate the specific mechanisms through which the immunoproteasome and protein accumulation resulting from proteasome dysfunction may contribute to corneal thinning and the structural changes characteristic of keratoconus. Such research could provide valuable insights into potential therapeutic strategies for addressing these pathological alterations.

In ocular surface diseases, including dry eye and keratoconus, effective management is crucial for enhancing visual quality. However, current treatments often fall short of expectations, primarily focusing on alleviating symptoms while neglecting underlying factors such as oxidative stress, inflammation, and protein accumulation, which significantly contribute to the pathophysiology of these diseases. Therefore, future research should investigate the role of the UPS in disease biology, as a comprehensive understanding of its function could provide insights into more effective therapeutic strategies that target the root causes of these conditions.

### 3.2. Posterior Segment of the Eye

#### 3.2.1. Age-Related Macular Degeneration

Age-related macular degeneration (AMD) is one of the causes of global blindness [105]. Within its etiopathogenesis, there are various factors, including mutagenesis in some candidate genes, epigenetic factors, and lifestyle factors [106,107,108,109]. Biological events are related to protein degradation or PTMs that some proteins may undergo that maintain retinal homeostasis from visual development to retinal neurodegeneration processes [110].

The immunoproteasome plays a significant role in retinal function and health, particularly in the context of visual transmission [111,112]. For instance, immunoproteasomes are present in high concentrations in the photoreceptors and synaptic regions of the retina, suggesting their importance in visual transmission. Studies using immunoproteasome knockout (KO) mice have demonstrated that a deficiency in immunoproteasome subunits leads to defects in bipolar cell responses, resulting in a decrease in the amplitude of electroretinography (ERG) b-waves by approximately 25% [111]. This highlights their relevance in retinal physiology and physiopathology processes.

On the other hand, the immunoproteasome has been shown to provide cytoprotective effects in the retina under conditions of oxidative stress and injury [12]. For instance, retinal pigment epithelial cells lacking immunoproteasome components exhibited increased susceptibility to peroxide-induced cell death, indicating that the immunoproteasome plays a protective role against oxidative damage and its possible implication in age-related macular degeneration.

Considering that the retina has a high metabolic rate, rapid and efficient activities are required for the modification of proteins and, where appropriate, to lead them to degradation. However, as age advances and adds to other factors, such as UV exposure, poor diet, and cigarette intake [113], these degradation processes and modifications in proteins can alter in a more accelerated manner. Therefore, the accumulation of proteins in the macular area and its relationship with cellular senescence and aging can be explained by autophagy and UPS activity [114,115].

In an animal model, it has been demonstrated that inhibiting the activity of the proteasome results in the accumulation of oxidized proteins, including lipofuscin; this is a key component in the formation of the dominated “drusen” [3]. Therefore, there is an implication of proteasome activity. The proteasome not only focuses on protein degradation, but also how relevant processes, such as oxidative stress, cell signaling, inflammation, and key elements in the physiopathogenesis of AMD, can be regulated through the ubiquitination–proteasome pathway.

Consequently, Lee H. et al. reported that, in aqueous humor samples of individuals with AMD, analyzed by proteomics, a significant increase in proteins related to proteasome activity could be seen, including the regulatory subunit Rpn2 [116]. In addition, Qin et al., proposed that inhibiting the proteasome pharmacologically in RPE cells induced deregulation of the NF-κB pathway, which finally led to the differential expression of pro-inflammatory and proapoptotic genes, aspects that agree with what was reported by Fernandez A.F. et al. and Qin T. et al., who suggested that UPS has a role both in inflammation, and oxidative stress in AMD [117,118]. The above shows that the activity of the UPS influences many events in the retinal tissue and that this could be a critical element in the search for therapeutic interventions for AMD [114,119], considering its multiple roles in both retinal development and neurodegeneration.

#### 3.2.2. Glaucoma

Glaucoma is defined as a neurodegenerative disease that includes a variety of biological events including glutamate excitotoxicity, mitochondrial dysfunction, cholinergic deregulation, etc., which lead to loss of retinal ganglion cells (RGCs), clinically converted by the loss of the nerve fiber layer of the retina, in some cases with an increase in intraocular pressure (IOP) and, with these, changes in functions such as contrast sensitivity and visual field [73].

Recent studies reveal molecular mechanisms that include protein misfolding, protein aggregation, and the role of UPS activity in regulating protein degradation processes. An event that has been highlighted in the pathophysiology of neurodegenerative diseases.

Indeed, several studies support that glaucoma should not only be considered an eye disease, but it can also become a risk factor for Alzheimer’s disease [120,121]. In fact, the pathophysiological mechanisms of glaucoma converge with Alzheimer’s when comparing characteristics such as cholinergic deficiency, glutamate excitotoxicity, and neuroinflammation [73]. For instance, Korac J et al. (2013) demonstrated the role of ubiquitination in the formation of misfolded proteins in different neurodegenerative diseases such as Alzheimer’s, Parkinson’s, and amyotrophic lateral sclerosis (ALS) [122].

Optineurin (OPTN) is a protein that interacts with a series of events, including cell morphogenesis, vesicular trafficking, transcriptional regulation, and autophagy, and has been a genetic marker in normal tension glaucoma and primary open-angle glaucoma (POAG) [123,124]. Under physiological conditions, OPTN interacts with LC3 to degrade its substrates through autophagy [125]. Thus, when analyzing the protein structure, it can be seen that it contains a ubiquitin-binding domain, and studies such as that of Shen WC have shown that mutations in the region related to Ub recognition in the OPTN gene interfere with the process of autophagy, and, thus, OPTN colocalizes with misfolded proteins [126].

Conversely, Kageyama et al. indicated that chemical inhibition of the proteasome leads to a reduction in proteasome activity and an increase in the level of polyubiquitinated proteins, ultimately resulting in retinal degeneration and the death of photoreceptors and retinal ganglion cells [127]. Similar findings were reported by Hayat B et al. in patients with pseudoexfoliation glaucoma, who exhibited increased expression of ER-stress markers and decreased proteasome activity [128]. This imbalance in proteasome function underscores its clinical significance and highlights the UPS as a promising therapeutic target in retinal diseases such as glaucoma.

From another point of view, inflammation has been considered a possible etiology for the release of inflammatory mediators from trabecular meshwork (TM) cells [73,129]. Indeed, studies of tear proteomes and aqueous humor samples from glaucoma patients demonstrate a pro-inflammatory profile, supporting the role of inflammation in glaucoma [130]. Keller K.E. and Wirtz M.K. described that the *ASB10* gene is associated with the development of glaucoma and, interestingly, *ASB10* is regulated by pro-inflammatory cytokines that, in turn, contain the suppressor of cytokine signaling (SOCS) gene family that contains ubiquitination domains and could recruit complexes of Ub ligase for subsequent degradation by the proteasome [131].

In studies of POAG individuals with elevated IOP levels, differential expression of *ASB10* has been identified; it has a role in regulating aqueous humor outflow and maintaining IOP homeostasis [132]. Although direct involvement of the immunoproteasome in glaucoma pathology has not been thoroughly investigated, its established role in other neurodegenerative conditions and its significance in regulating inflammation position it as a promising candidate for further study.

Consequently, Keller K.E. et al. (2013) demonstrated that TM cells express ASB10 and contain motifs facilitated by two proteins: Cullin5 and elonginBC. This study suggests that ASB10 may function as a Ub ligand, promoting the degradation of specific proteins in TM cells. This mechanism is crucial for understanding of the regulation of cellular homeostasis and provides insights into glaucoma pathogenesis, where the accumulation of misfolded or excess proteins can lead to cellular dysfunction and impaired tissue function. [133].

The immunoproteasome has emerged as a potential therapeutic target in various neurodegenerative diseases, including Alzheimer’s disease and cardiovascular diseases [49,63]. In Alzheimer’s disease, increased immunoproteasome activity has been observed in reactive glial cells surrounding amyloid plaques, suggesting its involvement in disease pathogenesis [134]. Tezel et al. have proposed that proteasome activity could serve as a biomarker in human glaucoma models [135]. The immunoproteasome offers valuable insights into disease progression and holds promise as a therapeutic target. Its involvement in neurodegenerative diseases, along with its potential as a biomarker and treatment target, warrants further investigation, particularly in glaucoma. Exploring the immunoproteasome’s role in glaucoma pathophysiology could pave the way for novel treatment strategies targeting this proteasome subtype.

Age-related retinal degeneration represents a significant public health concern, given the increasing number of individuals in older age groups. Understanding the biological events occurring within the retina is crucial, as it could lead to early detection of changes primarily associated with protein accumulation and dysfunction in key biological systems, such as the UPS. This knowledge may pave the way for more effective diagnostic and therapeutic strategies aimed at mitigating the impact of retinal degeneration in aging populations.

## 4. Treatments Targeting the Proteasome in Ocular Pathologies

The proteasome has garnered significant attention as a potential therapeutic target in various diseases [136]. Recent advances in understanding the role of proteasomal dysfunction in the pathogenesis of multiple conditions have led to the exploration of novel drug development strategies focused on modulating proteasomal activity [137].

In the context of ocular diseases, where protein homeostasis is crucial for maintaining cellular integrity, proteasome-targeted therapies hold great promise. Diseases like keratoconus, dry eye, glaucoma, and age-related macular degeneration (AMD) have been linked to impaired protein degradation, leading to the accumulation of misfolded or dysfunctional proteins [138]. These accumulated proteins can trigger cellular stress, inflammation, and tissue damage, which ultimately contribute to disease progression. Therefore, restoring proper proteasomal function could be a pivotal strategy in preventing or even reversing disease processes.

The development of small molecules or biologics that either enhance or inhibit proteasomal activity offers a promising avenue for therapeutic intervention. Proteasome activators could help with improving protein degradation, especially in diseases characterized by the accumulation of damaged proteins [137]. Conversely, proteasome inhibitors could be utilized to selectively degrade or to induce cell death in cancer cells where excessive protein degradation may be beneficial [139,140].

Targeting the proteasome holds promise for developing more specific and effective therapies, potentially improving patient outcomes and transforming the management of ocular diseases. Further investigation into the precise mechanisms underlying proteasomal regulation and the optimal modulation of its activity will be crucial to fully unlock the therapeutic potential of this approach. Currently, proteasome inhibitors are being explored in the contexts of cancer, autoimmunity, and viral infections, and this strategy could potentially be adapted as a novel therapeutic approach for ocular diseases (Table 1).

## 5. Conclusions and Future Perspectives

The eye is a target organ for many biological events, including oxidative stress, inflammation, and processes that are required by the UPS to maintain ocular homeostasis. However, due to various exogenous and endogenous factors, this balance can be lost, generating an accumulation of protein components that can trigger responses that favor the appearance of diseases such as dry eye, glaucoma, AMD, and others, which is why, in the last decade, a significant number of publications have focused their attention on understanding what happens with “cellular proteostasis” and how, from the loss of proteostasis, pathways can appear that lead to the development of the disease [8,151,152].

This highlights the critical role of the UPS in regulating the activity and maintenance of numerous proteins, either through ubiquitination signals or by directing them for degradation via proteasomal pathways. The involvement of the UPS in ocular health provides valuable insights not only into the pathophysiology of ocular diseases but also in identifying potential biomarkers associated with UPS dysfunction. For example, retinal degenerative diseases are frequently marked by the accumulation of misfolded proteins and protein aggregation, which are key pathological features [153]. These insights open the door to exploring pharmacological interventions targeting proteasome in the treatment of eye diseases.

Although numerous studies are included in this review concerning the role of the UPS, several limitations exist regarding the therapeutic approaches to the ocular pathologies described. While the UPS is indeed involved in protein accumulation and recycling, further research is essential to determine whether specific E3 ligases are associated with protein buildup in ocular tissues, and to explore whether the proteasome and immunoproteasome could serve as viable therapeutic targets.

Additionally, the UPS plays a crucial role in the removal of damaged or misfolded proteins, and its dysfunction can lead to the accumulation of toxic proteins, resulting in inflammation, cell death, and further vision impairment. Investigating the molecular mechanisms underlying UPS failure in retinal cells could provide valuable insights into potential strategies for targeting this system to slow or prevent disease progression.

Currently, within pharmacological interventions for many diseases such as cancer, autoimmune and neurodegenerative disease, have been described as either activating or inhibiting the activity of the proteasome [137,154,155,156]. Therefore, considering the implications of UPS activity on various ocular structures, one can think of future and novel therapeutic approaches to various ocular disorders.

In fact, medications based on proteasome inhibitors have already been patented for the treatment of dry eye [157], which shows that they can be a therapeutic line to explore for many diseases of both the anterior and posterior segments of the eye.

In comparison to other conditions, such as neurodegenerative disorders, autoimmune conditions, infectious diseases, and cancer, the investigation of the role of the UPS in ophthalmology remains in its early stages. Nonetheless, this progress has been facilitated by a deeper understanding of the biological events associated with ocular diseases, supported by insights from omics approaches, in vitro assays, and other methods. These efforts have enabled the identification of disrupted metabolic pathways and have clarified the relationship between the UPS and additional biological pathways that maintain cellular homeostasis. Consequently, there remains significant scope for further research and development in this area for application in the field of ophthalmology.

## Figures and Tables

**Table 1:** Treatments targeting the proteasome in ocular pathologies.

Treatment	Mechanism of Action	Ocular Pathologies	Outcome/Effect	Refs.
Bortezomib	Inhibits the 26S proteasome, preventing protein degradation	Wet AMD, diabetic retinopathy	Reduces inflammation, prevents retinal cell death, and exerts a neuroprotective effect.	[141,142]
Carfilzomib	Inhibits the proteasome by binding to its catalytic subunits	Glaucoma, retinal degeneration	Reduces oxidative stress, and prevents retinal damage.	[143]
Lactacystin	Inhibits proteasomal activity by binding to the proteasome catalytic site	AMD	May modulate inflammatory pathways, retinal aging, and cell survival.	[127,144]
MG132	A potent, reversible proteasome inhibitor	Corneal injury, keratoconjunctivitis sicca.	Reduces cellular apoptosis, and promotes tissue repair.	[145,146]
		Glaucoma	MG132 could enhance the efficiency of transduction by a FIV vector expressing green fluorescent protein (GFP) in immortalized human trabecular meshwork cells.	[147]
Epoxomicin	Selectively inhibits the 20S proteasome, blocking protein degradation	AMD, Glaucoma	Suppresses pro-inflammatory signaling, mitigates retinal cell death, and promotes anti-inflammatory activity.	[148,149]
Ubistatin	Inhibits the ubiquitin-proteasome pathway	Retinal pathology	Enhances retinal neuron survival and may promote neurite growth and synaptogenesis.	[150]

All findings presented in this table are based on studies of proteasome inhibitors. FIV: feline immunodeficiency virus. AMD: Age-related macular degeneration. GFP: green fluorescent protein.

## Data Availability

Data are contained within the article.
